# Sibling Bullying in Middle Childhood is Associated with Psychosocial Difficulties in Early Adolescence: The Case of Individuals with Autism Spectrum Disorder

**DOI:** 10.1007/s10803-019-04116-8

**Published:** 2019-07-22

**Authors:** Umar Toseeb, Gillian McChesney, Jeremy Oldfield, Dieter Wolke

**Affiliations:** 1 Department of Education, Derwent College, University of York, York, YO10 5DD UK; 2 Department of Psychology, Manchester Metropolitan University, Brooks Building, 53 Bonsall Street, Manchester, M15 6GX UK; 3 Department of Psychology, University of Warwick, Coventry, CV4 7AL UK

**Keywords:** Sibling bullying, Psychosocial, Social, Emotional, Adolescence, Longitudinal

## Abstract

Sibling bullying is associated with various psychosocial difficulties. We investigated this in 231 individuals with Autism Spectrum Disorder (ASD) and 8180 without ASD between middle childhood (age 11 years) and early adolescence (age 14 years). On the whole, self-reported sibling bullying decreased from middle childhood to early adolescence. Despite this, individuals with ASD continued to report more sibling bullying as both perpetrator and victim in early adolescence than those without ASD. We found that self-report sibling bullying in middle childhood was associated with psychosocial difficulties in early adolescence. Moreover, individuals with ASD were more likely to report being bullied by both siblings and peers in middle childhood and this pattern of victimisation was associated with concurrent and longitudinal psychosocial difficulties.

**Electronic supplementary material:**

The online version of this article (10.1007/s10803-019-04116-8) contains supplementary material, which is available to authorized users.

In the UK, approximately 85% of children have at least one sibling (Tippett and Wolke 2015). Good quality sibling relationships are important as they help children to develop social skills and are a source of emotional support (Brown et al. 1996; Downey and Condron 2004; Stormshak et al. 1996). However, sibling relationships can also include frequent conflict and aggression. Up to 50% of children have been the victim of bullying by their siblings and around 40% of siblings have reported being the perpetrators of these bullying incidences (Wolke et al. 2015). Sibling bullying is defined as “any unwanted aggressive behaviour(s) by a sibling that involves an observed or perceived power imbalance and is repeated multiple times or is highly likely to be repeated; bullying may inflict harm or distress on the targeted sibling, including physical, psychological, or social harm” (Wolke et al. 2015, p. 918). It is surprising that compared to peer bullying, sibling bullying has been neglected in research. Although sibling bullying occurs often in front of the parents, with 55% of victims and 40% of perpetrators reporting that at least one parent (if not both) was present when the bullying occurred (Skinner and Kowalski 2013), it has been normalised by parents and researchers (Eriksen and Jensen 2009).

A few studies have explored the psychosocial correlates of sibling bullying in general population samples (Tucker et al. 2013, 2014a, b; van Berkel et al. 2018; Wolke and Samara 2004). These studies mostly used cross-sectional or retrospective methods, with only a limited number using a longitudinal approach (Bowes et al. 2014; Dantchev and Wolke 2018; Dantchev et al. 2018; van Berkel et al. 2018; Wolke et al. 2015). Despite this paucity in research, the few longitudinal studies that do exist suggest a strong dose–response relationship between sibling bullying at a young age and psychosocial difficulties in later life. In a UK based community cohort, children who reported being bullied by siblings several times a week when they were 12 years old were twice as likely to have depression, anxiety, and to self-harm at age 18 (Bowes et al. 2014). They were also three times more likely to have psychotic disorder at age 18 years, compared to those who reported less frequent or no sibling bullying (Dantchev et al. 2018). These longitudinal studies also indicated that sibling bullying often co-occurs with peer bullying and that the adverse effects on psychosocial difficulties were increased further if the children experienced both (Dantchev and Wolke 2018; Dantchev et al. 2018). To the best of the our knowledge, only two studies have investigated sibling bullying involvement in children with developmental disorders, such as those with Autism Spectrum Disorder (Toseeb et al. 2018) or Attention Deficit Hyperactivity Disorder (Tucker et al. 2017), groups who tend to have poor psychosocial outcomes.

Autism Spectrum Disorder (ASD) is a pervasive developmental disorder affecting ~ 1% of the population within the UK (Baird et al. 2006), although some argue that this might be a conservative estimate (Russell et al. 2014). ASD is characterised by social and communication difficulties, repetitive behaviours, and high sensitivity to sensory stimuli (American Psychiatric Association 2013). Children with ASD, experience higher levels of social and emotional difficulties compared to neurotypical children (Volkmar et al. 2014). Many children with ASD also experience difficulties in social interactions, such as turn taking in conversation, and deficits in non-verbal communication (American Psychiatric Association 2013). Such difficulties may have implications for their relationships with other children.

For neurotypical children, during adolescence the reliance on parental resources decreases and friendships become increasingly important and meaningful. The emerging importance of friendships during this critical period of adolescence is thought to have effects on subsequent behaviour (Wilkinson 2008). Therefore, it may be that, for neurotypical individuals, the levels of sibling bullying involvement decreases during adolescence as the importance of friendships becomes more salient and thus less emphasis is placed on sibling relationships. The pattern of development may be different for children and adolescents with ASD. It is well documented that adolescents with ASD have problems with friendships and they are more likely than those without ASD to be bullied by their peers (Sterzing et al. 2012). Therefore, it might be that friendships for children with ASD do not become more meaningful as they develop into adolescence and so the levels of sibling bullying involvement remain the same.

Many studies have investigated sibling bullying in neurotypical children and also peer bullying amongst children with ASD, but there is a paucity of research looking specifically at sibling bullying in children with ASD. To the best of our knowledge, only one study has previously investigated sibling bullying in children with ASD (Toseeb et al. 2018). In this study, ASD status was determined using parental reports as part of a wider UK based population cohort study. The researchers found that children with ASD were more likley to report being bullied by their siblings compared to children without ASD. They were also more likely to report being invovled in two-way sibling bullying, as both victim and perpetrator, compared to those children without ASD. In this cross-sectional study, two-way sibling bullying involvement was associated with internalising and externalising problems and lower levels of prosocial behaviour. These findings suggest that sibling bullying is an area of concern for children with ASD and warrants further investigation and replication.

The evidence base for negative outcomes associated with sibling bullying is building. In neurotypical samples, there is a strong dose–response longitudinal relationship between sibling bullying and psychosocial difficulties. For children with ASD, there is evidence for concurrent associations between sibling bullying involvement and psychosocial functioning but the longitudinal evidence is non-existent. We sought to address this gap in the literature by presenting three research questions. First, how does sibling bullying involvement change between the ages of 11 (middle childhood) and 14 years (early adolescence) for individuals with and without ASD (Research question 1)? Second, what are the psychosocial outcomes of sibling bullying for individuals with and without ASD, which we investigated prospectively over a 3-year period (Research question 2)? And third, what are the effects of being victimized in multiple contexts, i.e. by peers and siblings, on psychosocial outcomes for those with and without ASD (Research question 3)?

## Method

### Study Sample

The Millennium Cohort Study (MCS) is a multi-disciplinary study, which follows the lives of approximately 19,000 children born in the UK between the years 2000 and 2001 (University of London 2017a, b, c, d, e, 2018). Data was accessed via the UK Data Service (http://www.ukdataservice.ac.uk/). The Centre for Longitudinal Studies, UCL Institute of Education, the UK Data Archive, and UK Data Service bear no responsibility for the analysis or interpretation of these data.

The MCS sample population was randomly selected from UK electoral wards, with the application of disproportionate stratification in order to provide an adequate representation of all four areas of the UK (England, Scotland, Wales, Northern Ireland), including deprived areas and areas where there is a high concentration of ethnic minority families. Drawn from the entire live birth cohort of the UK between the years 2000 and 2001, the first data sweep was carried out when the children were 9 months old. At the time of writing, six data sweeps were available. Data was collected when children were aged 9 months (N = 18,522), 3 years (N = 15,590), 5 years (N = 15,246), 7 years (N = 13,857), 11 years (N = 13,287), and 14 years old (N = 11,726). MCS participants at each data sweep were surveyed on an extensive range of information, including parenting, cognitive development, education, and socioeconomic status. Full details of the MCS, including methodological information, is reported elsewhere (Connelly and Platt 2014). Data used in this paper were collected from cohort members (the children) and the primary caregiver, who was usually a parent.

In a number of cases, more than one child per household was surveyed. Only families with one child in the study were included in the analyses undertaken here. In addition, the following were also excluded: children with no siblings, those for whom ASD status could not be determined, and those who had missing sibling bullying data at either age 11 or 14 years. As described in the subsequent paragraphs, each child was assigned to only one of two mutually exclusive groups (with ASD or without ASD). Data was collected from parents and one child but not the siblings. Therefore, no information about the siblings, such as ASD diagnosis status, was available to include in the analyses. The total sample size after exclusions was 8411 (51% male).

#### Individuals With and Without Autism Spectrum Disorder (ASD)

The sample of individuals with ASD was determined using the process previously described by Toseeb et al. (2018). At parental interviews carried out when the child was 5, 7, 11, and 14 years old, the primary caregiver was asked “Has a doctor or health professional ever told you that (child) had Autism, Asperger’s syndrome or autistic spectrum disorder?” Those whose parents answered affirmatively to the question *at least one of the four time points* were included in the sample of “individuals with ASD”. Those whose parents answered *yes* at an earlier but *no* at a later time point (n = 118), were excluded from the sample of individuals with ASD and re-included in the sample of individuals without ASD. This yielded a sample size of 231 individuals with ASD (78% male). The remainder of the total sample will be subsequently referred to as “individuals without ASD”. The sample size of individuals without ASD was 8180 (50% male).

### Measures

#### Predictor Variables

##### Self-Report Sibling Bullying

When the child was 11 and 14 years old, he/she was asked to respond to two questions on a six-point scale (never, less often, every few months, approximately once a month, approximately once a week, most days): “how often do your brothers or sisters hurt you or pick on you on purpose?” (victimisation) and “how often do you hurt or pick on your brothers or sisters on purpose?” (perpetration). Based on previous work (Dantchev and Wolke 2018, 2019; Wolke and Samara 2004), three mutually exclusive sibling bullying groups were then defined as follows: victim-only: victimised at least once a week but not perpetrated at least once a week; bully-only: perpetrated at least once a week but not victimised at least once a week; bully-victim: both perpetrated and victimised at least and once a week. The correlations between a one item scale, such as the one used here, and multi-item scales was calculated in an independent sample [the Avon Longitudinal Study of Parents and Children (Boyd et al. 2013; Fraser et al. 2013)], and it was shown to be high (victimisation: *r* = 0.91, *n *= 6909, *p *< 0.01; perpetration: *r*_*pb*_ = 0.85, *n* = 6856, *p* < 0.01). Thus, there is good evidence for the validity of this short scale which was adopted in this multi-purpose cohort study.

##### Self-Report Peer Bullying

When the child was 11 years old, he/she was asked to respond to the following question: “how often do other children hurt you or pick on you on purpose?” (victimisation). Responses were coded on a six-point scale (never, less often, every few months, approximately once a month, approximately once a week, most days). Based on previous work (Dantchev et al. 2018), children were assigned to the peer bullying victim group if they were victimised by peers at least once a week.

#### Parent-Report Psychosocial Outcomes

The primary caregiver completed the Strengths and Difficulties Questionnaire, (SDQ, Goodman 1997) when the child was 11 and 14 years old. The items on the questionnaire were statements about their child. The primary caregiver was asked to respond on a three point scale the extent to which the statements applied to their child (not all, somewhat true, certainly true). The emotional and peer problems subscales were summed to create a measure of internalising symptoms (0 to 20). Conduct and hyperactivity subscales were summed to create a measure of externalising symptoms (0 to 20). The prosocial subscale was used to measure prosocial skills (0 to 10). Higher scores indicate more internalising symptoms, more externalising symptoms, and better prosocial skills. The internal reliability for all three measures was acceptable (age 11: internalising 0.75, externalising 0.80, prosocial skills 0.65, age 14: internalising 0.72, externalising 0.65, prosocial skills 0.73). The SDQ has previously been used to assess psychopathology in children with developmental disorders such as ASD and Developmental Language Disorder (Baird et al. 2006; Pickles et al. 2016). It has also been shown to be a valid screening tool for identifying mental health problems in children with cognitive, behavioural and developmental problems (Bryant et al. 2019).

#### Potential Confounders

##### Structural Family Variables

Primary caregivers completed a grid about other members of the household. This was used to determine lone parent status (one parent/carer or two parents/carers), number of siblings (1, 2, 3, 4 or more), and birth order (1st, 2nd, 3rd, 4th or later). Primary caregivers were asked to choose their child’s ethnicity from a list of options. A dummy variable was created (non-White or White). They were also asked to list income from all sources (e.g. main job, government benefits etc.), which was used to calculate their overall income. This was standardised using the OECD-modified scale (Hagenaars et al. 1994). Those families who were below the 60% median income level were categorised as low household income.

##### Individual Difference Variables

Sex of the child was determined at wave 1 assessment as female or male. To determine pre-existing psychopathology the primary caregiver completed the Strengths and Difficulties Questionnaire (SDQ, Goodman 1997) when the child was 3 years. The internal reliability for all three measures was acceptable (internalising 0.61, externalising 0.78, prosocial skills 0.66).

##### Cognition and Verbal Ability

At age 11 years, the verbal similarities subscale of the British Ability Scales (BAS, Elliot et al. 1996) was used to assess the child’s verbal ability. The BAS is a battery of tests which directly assesses the child’s cognitive ability. The format is as follows; The interviewer reads out a series of three words and the child is asked to say how the three words are related. Scoring instructions were used to generate standardised scores. Higher scores indicated better verbal ability. The Cambridge Neuropsychological Test Automated Battery (CANTAB) Spatial Working Memory Task (Robbins et al. 1994) was used as a proxy for cognitive function. The task is a touch-screen assessment which tests the child’s ability to retain spatial information and to manipulate remembered items in working memory. The total number of errors were used and reverse scored so that a higher score indicated better cognitive function. Both measures were used as indicative of wider cognitive function in the absence of a full battery of cognitive data being available. Scores on these two measures were standardised to generate z-scores.

##### Parent-Report Harsh Discipline

When the child was 5 years old, primary caregivers were asked to complete the Straus Conflict Tactics Scale (Straus and Hamby 1997) to assess harsh disciplining of their child. The scale consists of six items measuring how the primary caregiver deals with conflict with the child (e.g. how often child is shouted at when naughty). Responses were coded on a five-point scale [never, rarely, sometimes (~ once a month), often (~ once a week), daily]. Sum scores were generated (range 6 to 30). Higher scores indicated high rates of harsh discipline. The internal reliability of the measure was good (α = 0.71).

### Statistical Analyses

All analyses were conducted using Stata/SE 14.2 (StataCorp 2015) and two tailed tests, p < 0.05, were used. Confidence intervals were used in conjunction with the significance value to make inferences about statistical significance. To account for unequal sample attrition, the application of disproportionate stratification, and missing data, all estimates were weighted to population level (Mostafa 2014). All reported values are weighted estimates.

To address research question 1, multiple logistic regression models were run to investigate whether there was a change in the self-reported types of sibling bullying involvement between the two time points (11 and 14 years). For each model, the outcome was entered as the bullying involvement type (uninvolved, victim-only, bully-only, or bully-victim). The predictors were entered as age (11 or 14 years), ASD status (without ASD or with ASD), the interaction between age and ASD status. Sex, ethnicity, verbal ability, cognitive function, poverty, lone parent status, number of siblings, birth order, and harsh discipline were entered as covariates.

To address research question 2, multiple linear regression models were run to investigate whether self-reported sibling bullying involvement at age 11 years predicted psychosocial outcomes at age 14 years. Outcomes were either internalising problems, externalising symptoms, or prosociality. The predictors were self-reported sibling bullying involvement (uninvolved, victim-only, bully-only, bully-victim), ASD status (without ASD or with ASD), self-reported sibling bullying involvement × ASD status interaction. Sex, ethnicity, verbal ability, cognitive function, poverty, lone parent status, number of siblings, birth order, harsh discipline, and early psychopathology were entered as covariates.

To address research question 3, a new variable was created based on the child’s responses to the sibling and peer bullying victimisation questions at age 11 years. Children were assigned to three mutually exclusive “multi-victimisation” groups; uninvolved (picked on by sibling and peers less than once per week or never), sibling or peer victimisation (picked on by siblings *or* peers more than once per week), or sibling and peer victimisation (picked on by siblings *and* peers more than once per week). Multiple logistic regression models were run to compare whether membership of the three multi-victimisation groups was different based on ASD status (without ASD or with ASD). The dependent variables were entered as one of the following (uninvolved vs other two groups combined, sibling *or* peer victim vs other two groups combined, or peer *and* sibling victim vs other two groups combined). The predictor was entered as ASD status and the covariates were sex, ethnicity, verbal ability, cognitive function, poverty, lone parent status, number of siblings, birth order, and harsh discipline. Following this, the multiple regression models that were run for research question 2 were repeated, except that the independent variable was changed to multi-victimisation (peer *and* sibling victim vs other two groups combined).

## Results

### Change in Overall Levels of Sibling Bullying Involvement

**Fig. 1:**
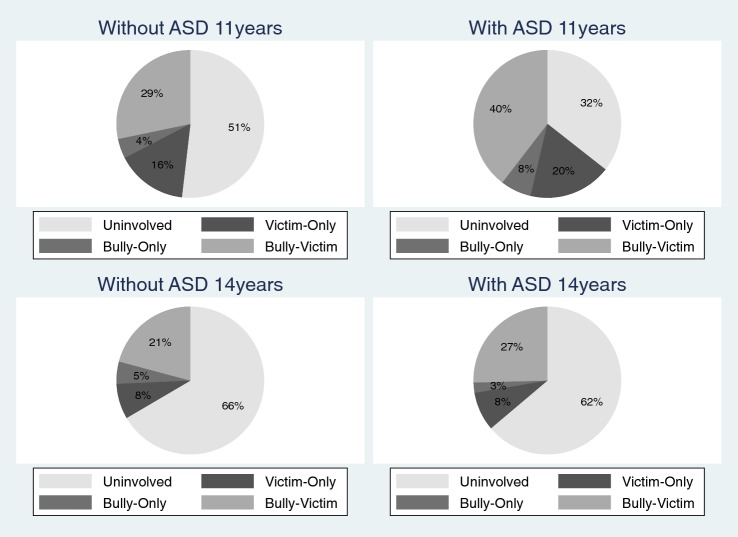
Breakdown of the different type of sibling bullying involvement by age and Autism Spectrum Disorder status

Descriptive statistics for the prevalence of self-reported sibling bullying involvement at age 11 and 14 years are shown in Table 1. Logistic regression models (Table 2) showed that, on the whole, the self-reported levels of sibling bullying involvement were different depending on age and ASD status. The confidence intervals for the interaction between age and ASD status warranted further investigation into the difference in change over time, separately for individuals with and without ASD. The odds of not being involved in sibling bullying increased between age of 11 and 14 for both individuals with and without ASD although, the odds were greater for indviduals with ASD. Moreover, self-reports showed that individuals with ASD were less likely than those without ASD to be uninvolved in any form of sibling bullying at the age of 11 but not at the age of 14. As shown in Fig. 1, when they were 11 years old, 32% of individuals with ASD reported that they were uninvolved in any sibling bullying (compared to 51% of children without ASD), which increased to 62% by the time they were 14 years old (compared to 66% of children without ASD). Therefore, the de-escalation in sibling bullying involvement between age 11 and 14 was greater for individuals with ASD compared to those without ASD. To this end, *by the time they reached age 14* *years, there was no difference in the overall levels of self*-*reported sibling bullying involvement between individuals with and without ASD.*

**Table 1:** Prevalence of sibling bullying at age 11 and 14 years according to ASD status, sex and household characteristics

	Age 11					Age 14				
	Total N (%)	Uninvolved (%)	Victim-only (%)	Bully-only (%)	Bully-victim (%)	Total N (%)	Uninvolved (%)	Victim-only (%)	Bully-only (%)	Bully-victim (%)
Overall	8411 (100)	4220 (50)	1356 (16)	370 (4)	2464 (29)	8411 (100)	5586 (66)	653 (8)	434 (5)	1739 (21)
ASD status										
Without ASD	8180 (100)	4146 (51)	1311 (16)	352 (4)	2371 (29)	8180 (100)	5443 (66)	633 (8)	427 (5)	1677 (21)
With ASD	231 (100)	75 (32)	45 (20)	18 (8)	93 (40)	231 (100)	142 (62)	19 (8)	7 (3)	62 (27)
Sex										
Boys	4273 (100)	2114 (49)	700 (16)	253 (6)	1207 (28)	4273 (100)	2964 (69)	308 (7)	275 (6)	725 (17)
Girls	4138 (100)	2106 (51)	657 (16)	117 (3)	1258 (30)	4138 (100)	2621 (64)	344 (8)	159 (4)	1013 (24)
Ethnicity										
Non-White	1361 (100)	813 (60)	167 (12)	60 (4)	231 (17)	1361 (100)	900 (66)	121 (9)	69 (5)	271 (20)
White	7050 (100)	3407 (48)	1189 (17)	310 (4)	2143 (30)	7050 (100)	4685 (66)	532 (8)	365 (5)	1467 (21)
Household income										
High	6396 (100)	3233 (51)	1015 (16)	252 (4)	1895 (30)	5663 (100)	3842 (68)	381 (7)	251 (4)	1188 (21)
Low	2015 (100)	987 (49)	341 (17)	118 (6)	569 (28)	2747 (100)	1743 (63)	271 (10)	183 (7)	550 (20)
Lone parent										
No	6415 (100)	3260 (51)	989 (15)	286 (4)	1880 (29)	6283 (100)	4234 (67)	466 (7)	293 (5)	1291 (21)
Yes	1996 (100)	960 (48)	367 (18)	84 (4)	584 (29)	2128 (100)	1352 (64)	187 (9)	141 (7)	448 (21)
Number of siblings										
1	4012 (100)	2142 (53)	545 (14)	158 (4)	1167 (29)	4185 (100)	2910 (70)	302 (7)	195 (5)	777 (19)
2	2619 (100)	1216 (46)	509 (19)	129 (5)	765 (29)	2489 (100)	1583 (64)	190 (8)	139 (6)	576 (23)
3	1197 (100)	568 (47)	204 (17)	55 (5)	369 (31)	1137 (100)	695 (61)	95 (8)	71 (6)	275 (24)
4 or more	583 (100)	294 (50)	97 (17)	28 (5)	163 (28)	601 (100)	397 (66)	64 (11)	29 (5)	110 (18)
Birth order										
1st	3016 (100)	1456 (48)	402 (13)	203 (7)	955 (32)	3016 (100)	1859 (62)	186 (6)	251 (8)	721 (24)
2nd	3076 (100)	1534 (50)	534 (17)	95 (3)	913 (30)	3076 (100)	2116 (69)	250 (8)	82 (3)	628 (20)
3rd	1285 (100)	690 (54)	216 (17)	36 (3)	342 (27)	1285 (100)	898 (70)	114 (9)	62 (5)	212 (16)
4th or later	675 (100)	355 (53)	136 (20)	23 (3)	161 (24)	674 (100)	464 (70)	72 (11)	26 (4)	113 (17)

**Table 2:** Predicting sibling bullying involvement as a function of age (11 and 14 years) and ASD status

	Odds ratio [95% CI]			
	Uninvolved	Victim-only	Bully-only	Bully-victim
Age	1.97 [1.83, 2.13]***	0.44 [0.39, 0.50]***	1.21 [0.97, 1.50]	0.62 [0.57, 0.68]***
With ASD	0.54 [0.36, 0.81]**	1.08 [0.62, 1.87]	1.44 [0.61, 3.40]	1.60 [1.03, 2.50]*
Age × ASD	1.61 [0.97, 2.68]	0.93 [0.39, 2.18]	0.24 [0.06, 1.04]	0.93 [0.49, 1.77]
Effect of age for children without ASD^a^	1.25 [1.22, 1.29]***	–	1.06 [0.99, 1.15]	–
Effect of age for children with ASD^a^	1.50 [1.24, 1.82]***	–	0.63 [0.41, 0.98]*	–
Effect of ASD at age 11^a^	0.58 [0.38, 0.86]**	–	1.49 [0.62, 3.59]	–
Effect of ASD at age 14^a^	0.83 [0.57, 1.21]	–	0.33 [0.09, 1.15]	–

There were four logistic regression models each with a different outcome variable: uninvolved (yes or no), victim-only (yes or no), bully-only (yes or no), or bully-victim (yes or no). All models included the predictors listed in the first column of the table and a number of covariates. The covariates have been omitted from this table for ease of comprehension but have been in included in the supplementary materials (Table S1)

^a^These are post hoc analyses which were run separately from the original logistics regression models only for those models where the confidence intervals were close to 1 for the age × ASD interaction

*p < 0.05, **p < 0.01, ***p < 0.001

### Changes in Specific Types of Sibling Bullying Involvement

#### Victim-Only

As shown in Table 2, the odds of self-reported involvement in sibling bullying as a victim-only decreased between age 11 and age 14. This effect was similar for individuals with and without ASD. When they were 11 years old, 20% of individuals with ASD (compared to 16% of individuals without ASD) were in the victim-only group, which decreased to 8% when they were 14 years old (compared to 8% of individuals without ASD). *That is, between age 11 and 14* *years, there was a decrease in the self*-*reported levels of sibling bullying involvement as a victim*-*only for both groups but there was no difference in the magnitude of the decrease between individuals with and without ASD.*

#### Bully-Only

In terms of self-reported sibling bullying involvement as a bully-only, overall there was no change between the ages of 11 and 14 years. When they were 11 years old, 8% of individuals with ASD (compared to 4% of individuals without ASD) were in the bully-only group, which decreased to 3% (compared to 5% of individuals without ASD) when they were 14 years old. The confidence intervals for the interaction between age and ASD status warranted further investigation of this effect using post hoc analyses (see Table 2). There was some indication that the proportion of bully-only involvement in individuals with ASD reduced between age 11 (n = 18) and 14 (n = 7), but this effect did not remain after correcting for multiple testing.

#### Bully-Victim

Overall, the odds of reporting sibling bullying involvement as a bully-victim decreased between age 11 and 14 years. At both time points individuals with ASD had increased odds of being involved in sibling bullying as a bully-victim compared to individuals without ASD. There was, however, no significant difference in the magnitude of the difference between the groups at age 11 or age 14 or in the rate of change between age 11 and 14. When they were 11 years old, 40% of individuals with ASD were in the bully-victim group (compared to 29% of individuals without ASD), which decreased to 27% when they were 14 years old (compared to 21% of individuals without ASD). Therefore, *although there was a reduction in self*-*reported sibling bullying involvement as bully*-*victim for individuals with and without ASD, the difference between the groups remained; individuals with ASD were more likely to report being involved in two*-*way sibling bullying, as a perpetrator and a victim.*

### Prospective Psychosocial Outcomes of Sibling Bullying

#### Internalising and Externalising Symptoms

As shown in Table 3, self-reports of being involved in any type of sibling bullying at age 11 were associated with higher internalising problems at age 14, when compared to those not reporting any sibling bullying at age 11. This effect was similar for individuals with and without ASD. Children who reported being involved in sibling bullying as a bully-only at age 11 had more externalising symptoms at age 14. Again, this effect was similar for individuals with and without ASD. Thus, *self*-*report sibling bullying involvement at age 11* *years was equally associated with internalising symptoms at age 14* *years for individuals with and without ASD*.

**Table 3:** Predicting psychosocial outcomes at age 14 years from sibling bullying role at age 11 years

	Internalising symptoms (14 years) Unstandardised beta [95% CI]	Externalising symptoms (14 years) Unstandardised beta [95% CI]	Prosocial skills (14 years) Unstandardised beta [95% CI]
Bullying involvement group (age 11)			
Uninvolved	0 [Reference]	0 [Reference]	0 [Reference]
Victim-only	0.28 [0.02, 0.53]*	− 0.11 [− 0.28, 0.06]	− 0.04 [− 0.20, 0.12]
Bully-only	0.54 [0.12, 0.96]*	0.36 [0.05, 0.67]*	− 0.34 [− 0.60, − 0.08]**
Bully-victim	0.32 [0.13, 0.51]**	0.04 [− 0.11, 0.19]	− 0.19 [− 0.33, − 0.05]*
ASD group			
Without ASD	0 [Reference]	0 [Reference]	0 [Reference]
With ASD	3.27 [1.94, 4.60]***	1.61 [0.40, 2.82]**	− 1.42 [− 2.09, − 0.76]***
Bullying involvement group × ASD group			
Uninvolved × ASD	0 [Reference]	0 [Reference]	0 [Reference]
Victim-only × ASD	0.22 [− 1.65, 2.09]	0.17 [-1.70, 2.05]	− 0.83 [− 2.00, 0.34]
Bully-only × ASD	− 2.18 [− 5.25, 0.89]	0.65 [-0.81, 2.11]	0.47 [− 2.72, 3.66]
Bully-victim × ASD	− 1.16 [− 2.76, 0.42]	− 0.95 [− 2.55, 0.65]	0.08 [− 0.89, 1.04]

There were three multiple regression models each with a differing outcome variable: internalising symptoms, externalising symptoms, or prosocial skills. All models included the predictors listed in the first column of the table and a number of covariates. The covariates have been omitted from this table for ease of comprehension but have been in included in the supplementary materials (Table S2)

*p < 0.05, **p < 0.01, ***p < 0.001

#### Prosocial Skills

Being a bully-only or bully-victim at age 11 was associated with lower prosocial skills at age 14 compared to those not reporting any involvement in sibling bullying at age 11. There was no difference in the effect for individuals with and without ASD. That is, *on the whole, children who reported being perpetrators of sibling bullying at age 11* *years (either as bully*-*only or bully*-*victim) were less prosocial when they were 14* *years old.*

### Victimisation Across Multiple Contexts

As shown in Table 4, at the age of 11 years old, children with ASD were more likely than children without ASD to report being victimised by both their siblings *and* their peers. This bullying in multiple contexts was investigated further to understand its associations with psychosocial outcomes both concurrently and prospectively. As expected, and shown in Table 5, children who reported that they were victimised in multiple contexts had more externalising symptoms (at age 11 and age 14 years) and lower prosocial skills (at age 11 and age 14 years) than those who did not report that they victimised in multiple contexts. There was no difference in these effects for individuals with and without ASD. For internalising symptoms, at age 11 years, the findings followed a different pattern as shown in the posthoc analyses of the interaction between bullying in multiple contexts X ASD group interaction. When those who reported that they were victimised in multiple contexts were compared to those who were not, individuals without ASD had more internalising symptoms, whilst individuals with ASD did not. That is, *on the whole, self*-*reports of being bullied by both siblings and peers were associated with worse psychosocial outcomes concurrently and prospectively compared to not being bullied by both siblings and peers, with the exception of concurrent internalising symptoms.*

**Table 4:** Prevalence and odds ratios of victimisation in multiple contexts at age 11 years

	Uninvolved	Victim of sibling OR peer bullying	Victim of sibling AND peer bullying
ASD status			
Without ASD (*n* = 8154)	4121 (50%)	3078 (38%)	955 (12%)
With ASD (*n* = 229)	65 (28%)	100 (44%)	64 (28%)
Odds ratio [95% confidence intervals]	0.46 [0.31, 0.69]***	1.24 [0.86, 1.79]	2.13 [1.27, 3.59]**

There were three logistic regression models each with a differing outcome variable: uninvolved (yes or no), victim or sibling or peer bullying (yes or no) or victim of sibling and peer bullying (yes or no). All models included ASD status and a number of covariates. The covariates have been omitted from this table for ease of comprehension but have been in included in the supplementary materials (Table S3)

*p < 0.05, **p < 0.01, ***p < 0.001

**Table 5:** Predicting concurrent (age 11 years) and longitudinal (age 14) psychosocial outcomes from multiple context victimisation group at age 11 years

	Internalising symptoms		Externalising symptoms		Prosocial skills	
	Unstandardised beta [95% CI]		Unstandardised beta [95% CI]		Unstandardised beta [95% CI]	
	Age 11	Age 14	Age 11	Age 14	Age 11	Age 14
Multiple contexts victim (Age 11)						
No	0 [Reference]	0 [Reference]	0 [Reference]	0 [Reference]	0 [Reference]	0 [Reference]
Yes	1.17 [0.84, 1.50]***	0.68 [0.36, 0.99]***	0.99 [0.68, 1.31]***	0.22 [0.00, 0.43]*	− 0.23 [− 0.37, − 0.08]**	− 0.30 [−0.51, − 0.09]**
ASD group						
Without ASD	0 [Reference]	0 [Reference]	0 [Reference]	0 [Reference]	0 [Reference]	0 [Reference]
With ASD	4.29 [3.35, 5.23]***	2.69 [1.87, 3.51]***	2.73 [1.97, 3.49]***	1.29 [0.57, 2.01]***	− 0.74 [− 1.15, − 0.33]***	− 1.30 [− 1.78, − 0.81]***
Multiple contexts victim (Age 11) × ASD	− 1.84 [− 3.75, 0.06]	− 0.42 [− 1.74, 0.91]	− 0.39 [− 2.29, 1.52]	− 0.33 [− 2.16, 1.51]	− 0.15 [− 1.24, 0.94]	− 0.63 [− 2.02, 0.77]
Non-victim (without ASD vs with ASD)^a^	4.27 [3.33, 5.22]***	–	–	–	–	–
Victim (without ASD vs with ASD)^a^	2.38 [0.60, 4.17]**	–	–	–	–	–
Without ASD (victim vs no victim)^a^	1.29 [0.95, 1.63]***	–	–	–	–	–
With ASD (victim vs no victim)^a^	0.80 [− 1.27, 2.87]	–	–	–	–	–

There were six multiple regression models each with a differing outcome variable: internalising symptoms age 11, internalising symptoms age 14, externalising symptoms age 11, externalising symptoms age 14, prosocial skills age 11, or prosocial skills age 11. All models included all the variables listed in the first column and a number of covariates. The covariates have been omitted from this table for ease of comprehension but have been in included in the supplementary materials (Table S4)

^a^These are post hoc analyses which were run separately from the original logistics regression models only for those models where the confidence intervals were close to zero for the age × ASD interaction

*p < 0.05, **p < 0.01, ***p < 0.001

## Discussion

### Change in Sibling Bullying Over Time

In this population based longitudinal cohort study, we found that the *overall levels* of self-reported sibling bullying involvement decreased between middle childhood and early adolescence. This is consistent with previous reports in the general population (Tucker et al. 2014b). In middle childhood, children with ASD are more likely than those without ASD to report being involved in any type of sibling bullying. By the time they reach early adolescence this difference no longer exists. That is, in early adolescence, those with ASD, on the whole reported similar levels of sibling bullying involvement as their peers without ASD (differences in the specific types of sibling bullying involvement still exist, which are discussed later). This is due to the greater self-reported de-escalation of sibling bullying involvement between middle childhood and early adolescence for individuals with ASD compared to those without ASD. It may be that the increasing importance of peers during adolescence (Wilkinson 2008) decreases the competition for parental resources. It is possible that the siblings of individuals with ASD may orientate themselves more outside the family and thus less conflict between siblings arises. Furthermore, research shows that the nature of bullying changes over the course of childhood development, with early adolescence seeing a rise in the role peers play in supporting and promoting bullying (Craig and Pepler 2003). On the whole, self-reported differences in sibling bullying involvement between individuals with and without ASD cease to exist by the time they reach early adolescence. That said, when focussing on two-way involvement in sibling bullying, as a victim and a preparator, even in early adolescence, those with ASD are more likely than those without ASD to report being involved in two-way sibling bullying.

There are a number of reasons why one might expect sibling bullying experiences to persist into adolescence for those with ASD. Social and communication difficulties may make individuals with ASD more prone to persistent sibling bullying involvement, indeed such difficulties are related to peer bullying in children with ASD (Cappadocia et al. 2012). Alternatively, sibling bullying may be more likely in families who have a child or adolescent with ASD due to a higher risk of poorer communication skills within these families. There is evidence for social impairment (Constantino et al. 2006), language difficulties (Toth et al. 2007), and poorer social-communicative interactions (Stoner et al. 2007) in siblings of children with ASD. This also extends to parents of children with ASD (Dawson et al. 2007). Therefore, the broader autism phenotype in family members might make undiagnosed siblings (i.e. those who have not been diagnosed with ASD but display signs) more likely to bully and subsequently it may exacerbate social difficulties experienced by children and adolescents with ASD.

Structural family-level factors may also be important from an evolutionary perspective where siblings are considered as natural born competitors for limited parental resources including affection, attention or material goods (Dantchev and Wolke 2019; Tanskanen et al. 2017). Children and adolescents with ASD might get priority access to these limited parental resources. This varying access may lead to conflictual competitive behaviour, such as sibling aggression, to develop (Archer 2013; Felson 1983). Data on siblings was not available for the current study and so we are unable to provide evidence for these interpretations. It may be that the factors that make individuals with ASD more likely to be involved in two-way sibling bullying during childhood persist and continue to have an effect during adolescence.

### Prospective Associations of Sibling Bullying Involvement

Our findings show that individuals with and without ASD, who report being involved in any type of sibling bullying in middle childhood, have higher levels of internalising symptoms in early adolescence compared to those not involved in any sibling bullying. These findings support previous work on the longitudinal effects of sibling bullying on psychosocial difficulties in the general population over and above pre-existing psychosocial difficulties and other household or parenting factors (Bowes et al. 2014; Dantchev et al. 2018). Our findings represent an important replication as we used an independent sample and different measures of psychosocial difficulties compared to previous work by Bowes et al. (2014) and Dantchev et al. (2018). This study adds that even in children and adolescents with a pervasive developmental disorder, sibling bullying is an additional environmental risk factor that is associated with an increase in symptoms of internalising disorder. Moreover, irrespective of ASD status, children who report being involved in sibling bullying as a perpetrator in middle childhood, have lower prosocial skills in early adolescence. These findings may be intuitive as the presence of some (but not all) bullying behaviours might indicate the absence of prosocial behaviours. For example, picking on a sibling (bullying behaviour) may mean that the child is not considerate of others’ feelings or kind to younger children (prosocial behaviours). This is not the case for all prosocial behaviours. For example, picking on a sibling (bullying behaviour) does not mean that the child does not share readily with other children (prosocial behaviour).

### Victimisation Across Multiple Contexts

Children with ASD are more likely than those without ASD to report being bullied by both their peers and their siblings. This suggests that, for some children with ASD, the vulnerabilities which make them susceptible to being victims of bullying are similar in both contexts. It does mean that when they return from school they have no respite from further victimisation at home. Moreover, the emotional difficulties experienced by children with ASD might make them the *perfect* victim (Zablotsky et al. 2013). In terms of externalising problems and prosocial skills, consistent with previous work in general population samples (Tucker et al. 2014b; Wolke and Skew 2012), being victimised in multiple contexts in middle childhood is associated with worse psychosocial outcomes both concurrently and longitudinally. This is similar for individuals with and without ASD. Therefore, the effects of multiple context victimisation on externalising problems and prosocial skills are similar for those with and without ASD. There was, however, an anomaly in terms of the findings for concurrent internalising problems. For children without ASD, but not those with ASD, being bullied in multiple contexts in middle childhood is associated with more internalising problems in early adolescence.

### Strengths, Limitations, and Implications

This study utilised of a large representative population-based sample. A clear advantage of this method is that accurate estimates of sibling bullying in individuals with and without ASD could be attained. This method has benefits over clinical population studies, which have been criticised for leading to inaccurate estimates due to issues with referral biases. By using a representative population-based sample, a number of potentially important variables were collected from each participants’ family. This enabled statistical models to include these additional variables as covariates and ensure that the effects observed were attributable to the variables investigated. It should be noted however, that residual confounding cannot be discounted.

Nonetheless, despite the strengths of this large representative sample and study design, there are some limitations that should be acknowledged. One limitation of this study is the use of parental-report to determine ASD status, rather than a clinical diagnosis. That said, parental-reports have consistently been used to estimate the prevalence of ASD (Boyle et al. 2011). Rises in the prevalence of ASD indicated through parental report are found to be similar to increases identified through clinical diagnoses (Van Naarden Braun et al. 2015). Whilst it is not a perfect indicator of a clinical diagnosis, the sensitivity of parental reports in identifying children with ASD have been found to be 95%, with specificity at 99% (Russell et al. 2015).

Given that children with ASD may have limited insight into the nature of social relationships, being unable to properly characterise and report experiences such as bullying may be problematic. However, research has shown that when parent and child reports of bullying experiences are compared, parental reports of bullying and victimization experiences for children with ASD were more in agreement than parental reports of typically developing offspring (Kloosterman et al. 2013). We take this as providing some evidence in support of using self-report measures of bullying in this study. That said, the use of self-report raises a further limitation. Those with poor literacy skills may have been excluded due to non-completion of the self-report questionnaire or they might have dropped out. Population and sample weights were utilised in order to minimise unequal attrition across the groups.

Whilst the use of self-report questionnaires is problematic, it is difficult to see a more reliable way to measure sibling bullying as it often occurs outside of the presence of parents. Perhaps in future studies adopting an approach that allows for independent corroboration of sibling bullying e.g. by the sibling, would help determine the extent to which this is a concern. Finally, the ASD status of the siblings was not accounted for here, which should be borne in mind when interpreting the findings.

These findings have important implications for the provision of resources for children and adolescents with ASD. Anti-bullying programmes specifically for those with ASD, whilst scarce, have proven effective. Video modelling techniques, in particular, have been used to teach children with ASD how to respond to bullying. For example, video modelling has taught children with ASD to make appropriate and assertive responses to bullying (Rex et al. 2018). The provision of more resources for children and adolescents with ASD could not only identify bullying behaviours and teach appropriate responses amongst their peers but could also be translated to the home.

## Conclusions

In this population-based sample of individuals with and without ASD, we found that, on the whole, self-reported levels of sibling bullying involvement decreased between middle childhood and early adolescence. Despite this overall decrease, those with ASD were still more likely to report being involved in two-way sibling bullying, as both a perpetrator and victim in early adolescence. Sibling bullying in middle childhood was associated with more internalising problems and lower prosocial skills in early adolescence irrespective of ASD status over and above other known risk factors. Moreover, children with ASD were more likely to report being bullied in multiple contexts (i.e. by their siblings and their peers) in middle childhood and this pattern of victimisation was associated with lower prosocial skills as well as more internalising and externalising problems in both middle childhood and early adolescence. If future studies are able to establish causation, a reduction in sibling bullying is likely to reduce the psychosocial difficulties for individuals with and without ASD.

## Electronic supplementary material

Below is the link to the electronic supplementary material.

Supplementary material 1 (DOCX 34 kb)
