# Supplementary material for: Sibling Bullying in Middle Childhood is Associated with Psychosocial Difficulties in Early Adolescence: The Case of Individuals with Autism Spectrum Disorder
Source: J Autism Dev Disord. 2019 Jul 22;50(5):1457–69. doi: 10.1007/s10803-019-04116-8 (PMC7211196; doi:10.1007/s10803-019-04116-8)
Supplement: Supplementary file 1 — Supplementary material 1 (DOCX 34 kb) [file 10803_2019_4116_MOESM1_ESM.docx]

**Supplementary Material**

Table S1.

*Predicting sibling bullying involvement by age (11 and 14 years) and ASD status*

|  | **Odds Ratio [95% CI]** | | | |
| --- | --- | --- | --- | --- |
|  | **Uninvolved** | **Victim-only** | **Bully-only** | **Bully-victim** |
| Age | 1.97 [1.82, 2.13]^***^ | 0.44 [0.39,0.50]^***^ | 1.21 [0.97, 1.50] | 0.62 [0.57, 0.68]^***^ |
| with ASD | 0.54 [0.36,0.81]^**^ | 1.08 [0.62, 1.87] | 1.44 [0.61, 3.40] | 1.60 [1.03, 2.50]^*^ |
| Age X ASD | 1.61 [0.97, 2.68] | 0.93 [0.39, 2.18] | 0.24 [0.06, 1.04] | 0.93 [0.49, 1.77] |
| Effect of age for children without ASD^a^ | 1.25 [1.22, 1.29]^***^ | - | 1.06 [0.99, 1.15] | - |
| Effect of age for children with ASD^a^ | 1.50 [1.24, 1.82]^***^ | - | 0.63 [0.41, 0.98]^*^ | - |
| Effect of ASD at age 11^a^ | 0.58 [0.38, 0.86]^**^ | - | 1.49 [0.62, 3.59] | - |
| Effect of ASD at age 14^a^ | 0.83 [0.57, 1.21] | - | 0.33 [0.09,1.15] | - |
| Female | 0.92 [0.84,1.01] | 0.95 [0.81,1.10] | 0.54 [0.44,0.68]^***^ | 1.32 [1.18,1.47] |
| White | 0.80[0.68, 0.94]^**^ | 1.21 [0.97,1.51] | 1.24 [0.88, 1.75] | 1.13 [0.94, 1.36] |
| Verbal Ability | 1.07 [1.02,1.13]^*^ | 0.99 [0.93,1.06] | 0.92 [0.82,1.04] | 0.94 [0.88,1.00]^*^ |
| Cognitive Function | 1.02 [0.98,1.07] | 0.99 [0.92, 1.06] | 0.98 [0.89,1.09] | 0.98 [0.93, 1.04] |
| Low Income | 0.88 [0.78,1.00]^*^ | 1.14 [0.92,1.42] | 1.71 [1.29,2.27]^***^ | 0.94 [0.81, 1.10] |
| Lone Parent Family | 0.87 [0.77,0.98]^*^ | 1.19 [0.95,1.49] | 0.99 [0.74,1.33] | 1.08 [0.94, 1.24] |
| Number of Siblings | 0.84 [0.78,0.89]^***^ | 1.04 [0.93,1.17] | 1.11 [0.97,1.27] | 1.18 [1.10, 1.27]^***^ |
| Birth Order | 1.17 [1.11,1.24]^***^ | 1.22 [1.12,1.32]^***^ | 0.64 [0.55,0.73]^***^ | 0.81 [0.76,0.86]^***^ |
| Harsh Discipline | 0.96 [0.95, 0.97]^***^ | 1.01 [0.99, 1.03] | 1.02 [0.99, 1.05] | 1.04 [1.03, 1.06]^***^ |

*p<.05, **p<.01, ***p<.001. ^a^These are post hoc analyses which were run separately from the original logistics regression models only for those models where the confidence intervals were close to 1 for the age X ASD interaction.

Table S2.

*Predicting psychosocial outcomes at age 14 years from sibling bullying role at age 11 years*

|  | **Internalising Symptoms (14y)**  **Unstandardised Beta [95% CI]** | **Externalising Symptoms (14y)**  **Unstandardised Beta [95% CI]** | **Prosocial Skills (14y)**  **Unstandardised Beta [95% CI]** |
| --- | --- | --- | --- |
|  |  |  |  |
| Bullying Involvement Group (Age 11) |  |  |  |
| Uninvolved | 0 [Reference] | 0 [Reference] | 0 [Reference] |
| Victim Only | 0.28 [0.02, 0.53]^*^ | -0.11 [-0.28, 0.06] | -0.04 [-0.20, 0.12] |
| Bully Only | 0.54 [0.12, 0.96]^*^ | 0.36 [0.05, 0.67]^*^ | -0.34 [-0.60, -0.08]^**^ |
| Bully-Victim | 0.32 [0.13, 0.51]^**^ | 0.04 [-0.11, 0.19] | -0.19 [-0.33, -0.05]^*^ |
| ASD Group |  |  |  |
| Without ASD | 0 [Reference] | 0 [Reference] | 0 [Reference] |
| With ASD | 3.27 [1.94, 4.60]^***^ | 1.61 [0.40, 2.82]^**^ | -1.42 [-2.09, -0.76]^***^ |
| Bullying Involvement Group X ASD Group |  |  |  |
| Uninvolved X ASD | 0 [Reference] | 0 [Reference] | 0 [Reference] |
| Victim Only X ASD | 0.22 [-1.65, 2.09] | 0.17 [-1.70, 2.05] | -0.83 [-2.00, 0.34] |
| Bully Only X ASD | -2.18 [-5.25, 0.89] | 0.65 [-0.81, 2.11] | 0.47 [-2.72, 3.66] |
| Bully-Victim X ASD | -1.16 [-2.76, 0.42] | -0.95 [-2.55, 0.65] | 0.08 [-0.89, 1.04] |
| Sex |  |  |  |
| Boys | 0 [Reference] | 0 [Reference] | 0 [Reference] |
| Girls | 0.77 [0.60, 0.94]^***^ | -0.07 [-0.19, 0.06] | 0.39 [0.28, 0.51]^**^ |
| Ethnicity |  |  |  |
| Non-White | 0 [Reference] | 0 [Reference] | 0 [Reference] |
| White | 0.40 [0.03, 0.77]^*^ | -0.07 [-0.29, 0.16] | 0.15 [-0.07, 0.38] |
| Verbal Ability | -0.23 [-0.32, -0.13]^***^ | -0.05 [-0.11, 0.02] | 0.06 [-0.00, 0.12] |
| Cognitive Function | -0.13 [-0.22, -0.04]^**^ | -0.05 [-0.11, 0.01] | 0.06 [-0.00,0.11] |
| Pre-existing Psychopathology^a^ | 0.24 [0.20, 0.29]^***^ | 0.09[0.07, 0.11]^***^ | 0.19 [0.15, 0.23]^***^ |
| Household Income |  |  |  |
| High | 0 [Reference] | 0 [Reference] | 0 [Reference] |
| Low | 0.48 [0.18, 0.78]^**^ | 0.53 [0.33,0.72]^***^ | -0.37 [-0.55, -0.19]^***^ |
| Lone Parent |  |  |  |
| No | 0 [Reference] | 0 [Reference] | 0 [Reference] |
| Yes | 0.43 [0.18, 0.68]^**^ | -0.07 [-0.28, 0.15] | -0.16 [-0.31, -0.01]^*^ |
| Number of Siblings | -0.00 [-0.13, 0.12] | 0.05 [-0.06, 0.16] | -0.15 [-0.23, -0.07]^***^ |
| Birth Order | 0.06 [-0.06, 0.17] | 0.10 [0.01, 0.18]^*^ | 0.19 [0.11, 0.27]^***^ |
| Harsh Parenting | 0.04 [-0.02, 0.07]^**^ | 0.04 [0.02, 0.06]^***^ | -0.06 [-0.08, -0.04]^***^ |

^+^p<.1, *p<.05, **p<.01, ***p<.001. ^a^Psychopathological scores at age 3. For internalising symptoms, early psychopathology refers to internalising symptoms in the SDQ at age 3. For externalising symptoms, early psychopathology refers to externalising symptoms in the SDQ at age 3. For prosocial skills, early psychopathology refers to prosocial scale of the SDQ at age 3.

Table S3.

*Prevalence and odds ratios of victimisation in multiple contexts at age 11 years*

|  | **Uninvolved** | **Victim of sibling OR peer bullying** | **Victim of sibling AND peer bullying** |
| --- | --- | --- | --- |
| ASD Status |  |  |  |
| Without ASD (n=8154) | 4121 (50%) | 3078 (38%) | 955 (12%) |
| With ASD (n=229) | 65 (28%) | 100 (44%) | 64 (28%) |
| Odds Ratio [95% Confidence Intervals] | 0.46 [0.31, 0.69]^***^ | 1.24[0.86, 1.79] | 2.13 [1.27, 3.59]^**^ |
| Female | 1.04 [0.93,1.17] | 0.99 [0.88, 1.12] | 0.93 [0.76, 1.14] |
| White | 0.65 [0.51, 0.83]^**^ | 1.32 [1.03, 1.69]^*^ | 1.50 [0.99, 2.27] |
| Verbal Ability | 1.15 [1.07, 1.22]^**^ | 0.95 [0.89, 1.01] | 0.83 [0.76, 0.92]^***^ |
| Cognitive Function | 1.07 [1.01, 1.13]^*^ | 0.95 [0.89, 1.01] | 0.97 [0.87, 1.06] |
| Low Income | 1.07 [0.91, 1.26] | 0.84 [0.70, 1.01] | 1.21 [0.91, 1.62] |
| Lone Parent Family | 0.80 [0.68, 0.95]^**^ | 1.14 [0.94, 1.38] | 1.24 [0.93, 1.67] |
| Number of Siblings | 0.85 [0.78, 0.92]^***^ | 1.11 [1.02, 1.21]^*^ | 1.14 [1.02, 1.27]^*^ |
| Birth Order | 1.08 [1.00, 1.16]^*^ | 0.96 [0.89, 1.03] | 0.93 [0.82, 1.05] |
| Harsh Discipline | 0.95 [0.93, 0.96]^***^ | 1.03 [1.01, 1.05]^***^ | 1.05 [1.03, 1.08]^***^ |

^+^p<.1, *p<.05, **p<.01, ***p<.001. ^a^Psychopathological scores at age 3. For internalising symptoms, early psychopathology refers to internalising symptoms in the SDQ at age 3. For externalising symptoms, early psychopathology refers to externalising symptoms in the SDQ at age 3. For prosocial skills, early psychopathology refers to prosocial scale of the SDQ at age 3.

Table S4.

*Predicting concurrent (age 11 years) and longitudinal (age 14) psychosocial outcomes from multiple context victimisation group at age 11 years*

|  | **Internalising Symptoms**  **Unstandardised Beta [95% CI]** | | **Externalising Symptoms**  **Unstandardised Beta [95% CI]** | | **Prosocial Skills**  **Unstandardised Beta [95% CI]** | |
| --- | --- | --- | --- | --- | --- | --- |
|  |  |  |  |  |  |  |
|  | **Age 11** | **Age 14** | **Age 11** | **Age 14** | **Age 11** | **Age 14** |
| **Multiple contexts victim (Age 11)** |  |  |  |  |  |  |
| No | 0 [Reference] | 0 [Reference] | 0 [Reference] | 0 [Reference] | 0 [Reference] | 0 [Reference] |
| Yes | 1.17 [0.84, 1.50]^***^ | 0.68 [0.36, 0.99]^***^ | 0.99 [0.68, 1.31] ^***^ | 0.22 [0.00, 0.43]^*^ | -0.23 [-0.37, -0.08] ^**^ | -0.30 [-0.51, -0.09]^**^ |
| **ASD Group** |  |  |  |  |  |  |
| Without ASD | 0 [Reference] | 0 [Reference] | 0 [Reference] | 0 [Reference] | 0 [Reference] | 0 [Reference] |
| With ASD | 4.29 [3.35, 5.23]^***^ | 2.69 [1.87, 3.51]^***^ | 2.73 [1.97, 3.49] ^***^ | 1.29 [0.57, 2.01]^***^ | -0.74 [-1.15, -0.33] ^***^ | -1.30 [-1.78, -0.81]^***^ |
| **Multiple contexts victim (Age 11) X ASD** | -1.84 [-3.75, 0.06] | -0.42 [-1.74, 0.91] | -0.39 [-2.29, 1.52] | -0.33 [-2.16, 1.51] | -0.15 [-1.24, 0.94] | -0.63 [-2.02, 0.77] |
| Non-victim (without ASD vs with ASD)^a^ | 4.27 [3.33, 5.22]^***^ | - | - | - | - | - |
| Victim (without ASD vs with ASD) ^a^ | 2.38 [0.60, 4.17]^***^ | - | - | - | - | - |
| Without ASD (victim vs no victim) ^a^ | -0.35 [2.43, 1.73] | - | - | - | - | - |
| With ASD (victim vs no victim) ^a^ | 1.17 [0.84, 1.51]^***^ | - | - | - | - | - |
| Female | 0.25 [0.05,0.44]^*^ | 0.77[0.61,0.94]^***^ | -0.68[-0.89,-0.48]^***^ | -0.07[-0.20,-0.25] | 0.43[0.33,0.54]^***^ | 0.39[0.27,0.51]^***^ |
| White | 0.10[-0.30,0.50] | 0.42[0.04,0.80]^*^ | -0.29[-0.11,0.69] | -0.01[-0.32,023] | 0.06[-0.13,0.25] | 0.14[-0.08,0.36] |
| Verbal Ability | -0.26[-0.37,-0.16]^***^ | -0.22[-0.31,-0.12]^***^ | -0.43[-0.55,-0.32]^***^ | -0.04[-0.11,0.02] | 0.08[0.02,0.13]^*^ | 0.05[-0.01,0.11] |
| Cognitive Function | -0.19[-0.28,-0.09]^***^ | -0.13[-0.23,-0.04]^**^ | -0.42[-0.51,-0.33]^***^ | -0.05[-0.11,0.01] | 0.02[-0.02,0.06] | 0.06[-0.00,0.11] |
| Pre-existing Psychopathology | 0.36[0.31,0.41]^***^ | 0.25[0.20,0.29]^**^ | 0.31[0.28,0.34]^***^ | 0.09[0.07,0.11]^***^ | 0.17[0.14,0.20]^***^ | 0.19[0.16,0.23]^***^ |
| Low Income | 0.47[0.09,0.86]^*^ | 0.46[0.16,0.77]^**^ | 0.82[0.42,1.21]^***^ | 0.54[0.34,0.74]^***^ | -0.26[-0.43,-0.09]^**^ | -0.36[-0.54,-0.18]^***^ |
| Lone Parent Family | 0.49[0.23,0.76]^***^ | 0.43[0.18,0.68]^**^ | 0.40[0.11,0.68]^**^ | -0.08[-0.30,0.03] | -0.14[-0.27,-0.01]^*^ | -0.15[-0.30,-0.00]^*^ |
| Number of Siblings | 0.03[-0.12,0.18] | -0.00[-0.13,0.12] | -0.03[-0.18,0.12] | 0.04[-0.07,0.15] | -0.11[-0.19,-0.03]^**^ | -0.15[-0.23,-0.07]^***^ |
| Birth Order | 0.07[-0.06,0.19] | 0.05[-0.06,0.17] | 0.10[-0.02,0.22] | 0.08[0.00,0.16]^*^ | 0.15[0.09,0.21]^***^ | 0.20[0.12,0.27]^***^ |
| Harsh Discipline | 0.09[0.07,0.12]^***^ | 0.04[0.02,0.07]^**^ | 0.21[0.18,0.23]^***^ | 0.04[0.02,0.05]^***^ | -0.06[-0.07,-0.04]^***^ | -0.06[-0.08,-0.04]^***^ |

^*^p<.05, ^**^p<.01, ^***^p<.001. There were six multiple regression models each with a differing outcome variable: internalising symptoms age 11, internalising symptoms age 14, externalising symptoms age 11, externalising symptoms age 14, prosocial skills age 11, or prosocial skills age 11. ^a^These are post hoc analyses which were run separately from the original logistics regression models only for those models where the confidence intervals were close to zero for the age X ASD interaction.
